# A Linear Analysis of Coupled Wilson-Cowan Neuronal Populations

**DOI:** 10.1155/2016/8939218

**Published:** 2016-09-20

**Authors:** L. L. Neves, L. H. A. Monteiro

**Affiliations:** ^1^Universidade Presbiteriana Mackenzie, Escola de Engenharia, São Paulo, SP, Brazil; ^2^Universidade de São Paulo, Escola Politécnica, São Paulo, SP, Brazil

## Abstract

Let a neuronal population be composed of an excitatory group interconnected to an inhibitory group. In the Wilson-Cowan model, the activity of each group of neurons is described by a first-order nonlinear differential equation. The source of the nonlinearity is the interaction between these two groups, which is represented by a sigmoidal function. Such a nonlinearity makes difficult theoretical works. Here, we analytically investigate the dynamics of a pair of coupled populations described by the Wilson-Cowan model by using a linear approximation. The analytical results are compared to numerical simulations, which show that the trajectories of this fourth-order dynamical system can converge to an equilibrium point, a limit cycle, a two-dimensional torus, or a chaotic attractor. The relevance of this study is discussed from a biological perspective.

## 1. Introduction

In the last decades, the features of action potentials propagating along axonal membranes have been related to the oscillations found in electroencephalogram (EEG) records [1–3]. From a theoretical perspective, analogies between EEG and nonlinear dynamics [4] were formulated prior to knowledge of the seminal numerical simulation by Lorenz, published in 1963, about a chaotic system [5]. All these studies aim to disclose the neural code; that is, to understand how information is represented, carried, and transformed by neurons [6]. The ambition is to explain how cognitive functions emerge from neuron spikes. In this context, here we analytically investigate the activity of a simple neuronal assembly, in order to examine how its parameter values influence its dynamical behavior.

Consider a population formed by two groups of interacting neurons, so that the synapses of the first group are excitatory and the synapses of the second group are inhibitory. Consider also that these two groups are connected. In 1972, Wilson and Cowan proposed a mathematical model to describe the time evolution of the activity of this neuronal population [7]. In this model, the activity *g*(*t*) of each group obeys a nonlinear first-order ordinary differential equation with the following form:

(1) $$\begin{array}{c} \frac{dg\left(t\right)}{dt}=-kg\left(\;t\;\right)+\left[\;1+rg\left(\;t\;\right)\;\right]S\left(\;z\left(\;t\;\right)\;\right) \end{array}$$

in which *t* denotes the time, *k* is a positive constant related to the natural decay of *g*(*t*), *r* is a positive constant proportional to the refractory period, and *z*(*t*) represents the input to this group, written as a linear combination of the activities of both groups plus the influence of external stimuli. Resting activity corresponds to *g* = 0; thus, a negative value of *g* expresses a depression from this background level. The function *S*(*z*) must be sigmoidal. However, as stated by Wilson and Cowan [7], “no particular significance is to be attached to the choice of” *S*(*z*). Usual choices are 1/(1 + *e* ^−*z*^) − 1/2 [8], tanh⁡(*z*) [9], and arctan⁡(*z*) [10]. In several studies, the properties of a single population were explored via numerical simulations [8, 9, 11], since these usual sigmoidal functions pose difficulties to analytical works. The dynamics of the two-population model was also already numerically investigated [10, 12–15].

Monteiro et al. suggested to use $S(z)=z/\sqrt{1+z^{2}}$ and some analytical results were derived for the Wilson-Cowan model [16]. Here, we also take this particular form of *S*(*z*) to analytically investigate the dynamics of two coupled Wilson-Cowan populations (composed of four groups). We also assume that (1) the isolated populations are identical (in the sense that they are characterized by the same parameter values, as considered in other studies [10, 12–15]); (2) the populations are coupled by links starting from their excitatory groups (because most synapses are excitatory; for instance, they are 84% in the cat cortex [17]); (3) the refractory period is negligible (which corresponds to taking *r* = 0 in (1), as supposed in several works [9, 10, 12, 14, 18–20]).

Some authors took *S*(*z*) as a piecewise linear function [14, 19–21]. In our analyses, we assume that the parameter values related to this neuronal assembly allow us to take *S*(*z*)≃*z*; thus, the model becomes linear. From this approximation, the occurrence of a Hopf bifurcation in the nonlinear model is analytically inferred. Other bifurcations are numerically found. Recall that bifurcation corresponds to a qualitative change in the dynamical behavior of a system caused by variation of parameter value(s).

This manuscript about the dynamics of a pair of coupled Wilson-Cowan neuronal populations is structured as follows. In Section 2, the model is presented and analyzed by taking into account the linear approximation just described. In Section 3, the analytical results are compared to numerical simulations performed by considering the nonlinear version of the model. We found that, as the time passes, the variables of the model can converge to an equilibrium point, a limit cycle, a two-dimensional torus, or a chaotic attractor. In Section 4, the results of this work are discussed from a biological point of view.

## 2. Analytical Results

Let two identical populations be coupled by links that can be symmetrical or asymmetrical. Assume that the variables *x*_*i*_(*t*) and *y*_*i*_(*t*) denote the activity of the excitatory group and the activity of the inhibitory group, respectively, of *i*th population, with *i* = 1,2. According to Wilson and Cowan, the dynamics of these coupled populations can be described by

(2) dx1tdt=−ax1t+wx1t−by1t+α1x2t+I11+wx1t−by1t+α1x2t+I12,dy1tdt=−dy1t+cx1t−ey1t+β1x2t+J11+cx1t−ey1t+β1x2t+J12,dx2tdt=−ax2t+wx2t−by2t+α2x1t+I21+wx2t−by2t+α2x1t+I22,dy2tdt=−dy2t+cx2t−ey2t+β2x1t+J21+cx2t−ey2t+β2x1t+J22.

**Figure 1:**
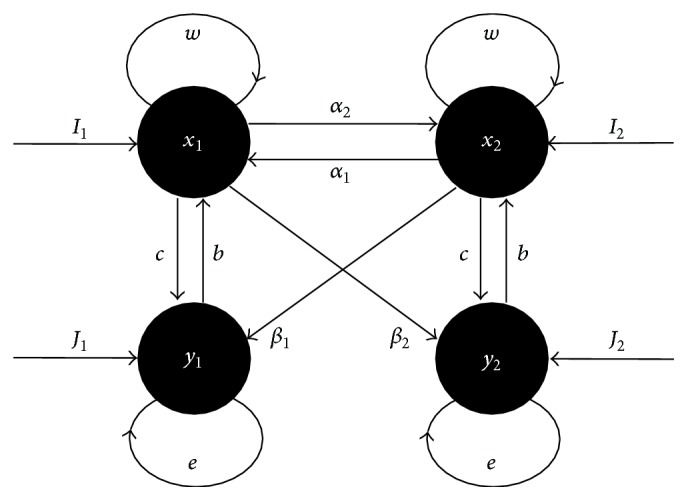
Schematic representation of the two-population model. The variables of the model are the activities of the neuronal groups *x*_1_, *x*_2_, *y*_1_, and *y*_2_. The connection strengths are denoted by *b*, *c*, *e*, *w*, *α*_1_, *α*_2_, *β*_1_, and *β*_2_. Stimuli from other sources correspond to *I*_1_, *I*_2_, *J*_1_, and *J*_2_.

The parameters *a* and *d* represent the natural (exponential) decay. The parameters *b*, *c*, *e*, and *w* are the strengths of the connections between the excitatory and inhibitory groups of an isolated population, as shown in Figure 1: *b* is the inhibitory connection from *y*_*i*_ to *x*_*i*_, *c* is the excitatory connection from *x*_*i*_ to *y*_*i*_, *w* is the self-excitatory connection (from *x*_*i*_ to *x*_*i*_), and *e* is the self-inhibitory connection (from *y*_*i*_ to *y*_*i*_). The strengths of the connections between the populations are denoted by the parameters *α*_*i*_ and *β*_*i*_: *α*_1_ is the connection from *x*_2_ to *x*_1_, *α*_2_ is the connection from *x*_1_ to *x*_2_, *β*_1_ is the connection from *x*_2_ to *y*_1_, and *β*_2_ is the connection from *x*_1_ to *y*_2_. All these ten parameters are positive constants. The constants *I*_1_, *J*_1_, *I*_2_, and *J*_2_ stand for stimuli from external sources reaching *x*_1_, *y*_1_, *x*_2_, and *y*_2_, respectively. Thus, the dimension of the parameter space is 14.

A steady state is a stationary solution, corresponding to an equilibrium point (*x*_1_^*∗*^, *y*_1_^*∗*^, *x*_2_^*∗*^, *y*_2_^*∗*^) in the four-dimensional state space *x*_1_ × *y*_1_ × *x*_2_ × *y*_2_. The constants *x*_1_^*∗*^, *y*_1_^*∗*^, *x*_2_^*∗*^, and *y*_2_^*∗*^ are obtained from *dx*_1_/*dt* = 0, *dy*_1_/*dt* = 0, *dx*_2_/*dt* = 0, and *dy*_2_/*dt* = 0. By taking into consideration the linear approximation proposed in Section 1, there is only one equilibrium point given by

(3) x1∗=bc−EWEI1−bJ1+EI2+bJ2Eα1−bβ1bc−EW2+Eα1−bβ1bβ2−Eα2,y1∗=bc−EWcI1−WJ1+EI2−bJ2cα1−Wβ1+bβ2−Eα2α1J1−β1I1bc−EW2+Eα1−bβ1bβ2−Eα2,x2∗=bc−EWEI2−bJ2+bJ1−EI1bβ2−Eα2bc−EW2+Eα1−bβ1bβ2−Eα2,y2∗=bc−EWcI2−WJ2+EI1−bJ1cα2−Wβ2+β2I2−α2J2Eα1−bβ1bc−EW2+Eα1−bβ1bβ2−Eα2,

with *W* ≡ *w* − *a* and *E* ≡ *d* + *e*. Note that if the populations are isolated, that is, if *α*_*i*_ = *β*_*i*_ = 0, then *x*_*i*_^*∗*^ = (*EI*_*i*_ − *bJ*_*i*_)/(*bc* − *EW*) and *y*_*i*_^*∗*^ = (*cI*_*i*_ − *WJ*_*i*_)/(*bc* − *EW*), for *i* = 1,2. Obviously, if *I*_1_ = *I*_2_ and *J*_1_ = *J*_2_, then *x*_1_^*∗*^ = *x*_2_^*∗*^ and *y*_1_^*∗*^ = *y*_2_^*∗*^.

The local stability of an equilibrium point can be determined from the eigenvalues *λ* of the Jacobian matrix obtained from the system of (2) linearized around this point [22–24]. By the Hartman-Grobman theorem, such a point is locally asymptotically stable if all eigenvalues have negative real parts [22–24]. For this fourth-order system, the eigenvalues *λ* are the roots of the polynomial:

(4) $$\begin{array}{c} \lambda^{4}+\gamma_{1}\lambda^{3}+\gamma_{2}\lambda^{2}+\gamma_{3}\lambda +\gamma_{4}=0, \end{array}$$

with

(5) γ1=2E−W,γ2=E−W2+2bc−EW−α1α2,γ3=2E−Wbc−EW+bα1β2+α2β1−2Eα1α2,γ4=bc−EW2+bEα1β2+α2β1−b2β1β2−E2α1α2.

Routh-Hurwitz criterion states that all eigenvalues have negative real parts if *γ*_1_ > 0, *γ*_2_ > 0, *γ*_3_ > 0, *γ*_4_ > 0, *δ*_1_ ≡ (*γ*_1_ *γ*_2_ − *γ*_3_)/*γ*_1_ > 0, and *δ*_2_ ≡ (*δ*_1_ *γ*_3_ − *γ*_1_ *γ*_4_)/*δ*_1_ > 0 [25]. For instance, in the case of isolated populations, local stability of the steady-state solution is assured if *E* > *W* and *bc* > *EW*.

Observe that the stability conditions do not depend on the values of *I*_*i*_ and *J*_*i*_. Thus, constant external stimuli do not modify the stability of the stationary solution (in the linear approximation). Observe also that a necessary condition for stability is *E* > *W*; that is, *w* < *w*_*c*_ ≡ *a* + *d* + *e* or *e* > *e*_*c*_ ≡ *w* − (*a* + *d*). Therefore, the equilibrium point is locally asymptotically stable only if the strength of the self-excitatory connection is below the critical value *w*_*c*_ and/or if the strength of the self-inhibitory connection is above the critical value *e*_*c*_.

If *α*_1_ = *α*_2_ = 0, then *γ*_4_ diminishes as *β*_1_ and/or *β*_2_ increase; if *β*_1_ = *β*_2_ = 0, then *γ*_2_, *γ*_3_, and *γ*_4_ diminish as *α*_1_ and/or *α*_2_ increase. Thus, both kinds of connections between the populations, characterized by *α*_*i*_ and *β*_*i*_, can singly destabilize the equilibrium point, if their strengths exceed critical numbers.

If the parameter values are varied so that *δ*_2_ = 0, then a Hopf bifurcation [22–24] can occur (because two roots of (4) are purely imaginary complex-conjugate numbers and the other two roots have negative real parts; i.e., (4) can be written as (*λ* ^2^ + *Aλ* + *B*)(*λ* ^2^ + *C*) = 0, with *A*, *B*, *C* > 0). As a consequence, a limit cycle with oscillation period $\tau ≃2\pi /\sqrt{C}$ appears. Recall that a limit cycle is an isolated closed trajectory in the state space, corresponding to a periodic solution [22–24].

If *α*_1_ = *α*_2_ = *α* and *β*_1_ = *β*_2_ = *β*, the condition *δ*_2_ = 0 is equivalent to *ϵ*_1_ ≡ (*w* + *α*)−(*a* + *d* + *e*) = 0. In this particular case, there is the birth of a limit cycle for *ϵ*_1_ = 0 if *ϵ*_2_ ≡ *b*(*c* + *β*)−(*w* + *α* − *a*)(*d* + *e*) > 0. The period *τ* of the newborn cycle is approximately given by $\tau ≃2\pi /\sqrt{\epsilon_{2}}$.

As shown in the next section, other bifurcations can take place by varying the parameter values of this dynamical system.

## 3. Numerical Simulations

In the simulations presented in this section, the values of *a*, *b*, *c*, *d*, *e*, *I*_1_, *I*_2_, *J*_1_, and *J*_2_ are kept fixed and the values of *w*, *α*_1_, *α*_2_, *β*_1_, and *β*_2_ are varied. Thus, we investigate how the dynamics is influenced by the strengths of the self-excitatory loops and of the links between the populations.

**Figure 2:**
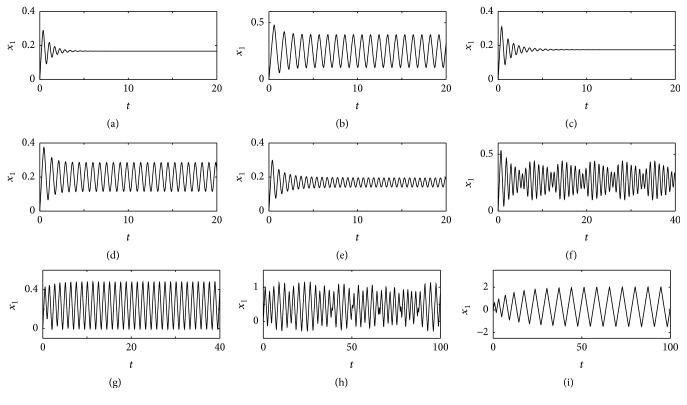
Temporal evolutions of *x*_1_(*t*) obtained by numerically integrating (2). In all cases, the initial condition is the origin of the state space. In all cases, *a* = *d* = 0.01, *b* = 20, *c* = 10, *e* = 10, *I*_1_ = 2, *I*_2_ = 1, and *J*_1_ = *J*_2_ = 0. In (a), *w* = 8, *α*_1_ = *α*_2_ = *β*_1_ = *β*_2_ = 0; in (b), *w* = 12, *α*_1_ = *α*_2_ = *β*_1_ = *β*_2_ = 0; in (c), *w* = 8, *α*_1_ = *α*_2_ = 1, and *β*_1_ = *β*_2_ = 0; in (d), *w* = 8, *α*_1_ = *α*_2_ = 3, and *β*_1_ = *β*_2_ = 0; in (e), *w* = 8, *α*_1_ = *α*_2_ = 1, *β*_1_ = 0, and *β*_2_ = 3; in (f), *w* = 12, *α*_1_ = *α*_2_ = 1, and *β*_1_ = *β*_2_ = 0; in (g), *w* = 12, *α*_1_ = *α*_2_ = 1, and *β*_1_ = *β*_2_ = 2; in (h), *w* = 13, *α*_1_ = *α*_2_ = 3, and *β*_1_ = *β*_2_ = 0; and in (i), *w* = 13, *α*_1_ = *α*_2_ = 3, and *β*_1_ = *β*_2_ = 2. In (a) and (c), the system converges to an equilibrium point; in (b), (d), (e), (g), and (i) to a limit cycle; in (f) to a two-dimensional torus; and in (h) to a chaotic attractor.

Here, we take *a* = *d* = 0.01, *b* = 20, *c* = 10, *e* = 10, *I*_1_ = 2, *I*_2_ = 1, and *J*_1_ = *J*_2_ = 0. In Figures 2(a)–2(i), only *x*_1_(*t*) is plotted in function of *t*. In each case, the behaviors of the other three variables are qualitatively similar; hence, their plots were omitted. For instance, if *x*_1_(*t*) converges to a periodic solution, then *y*_1_(*t*), *x*_2_(*t*), and *y*_2_(*t*) also tend to a periodic oscillation as the time passes. In (a), *w* = 8, *α*_1_ = *α*_2_ = *β*_1_ = *β*_2_ = 0; in (b), *w* = 12, *α*_1_ = *α*_2_ = *β*_1_ = *β*_2_ = 0; in (c), *w* = 8, *α*_1_ = *α*_2_ = 1, *β*_1_ = *β*_2_ = 0; in (d), *w* = 8, *α*_1_ = *α*_2_ = 3, and *β*_1_ = *β*_2_ = 0; in (e), *w* = 8, *α*_1_ = *α*_2_ = 1, and *β*_1_ = 0, *β*_2_ = 3; in (f), *w* = 12, *α*_1_ = *α*_2_ = 1, and *β*_1_ = *β*_2_ = 0; in (g), *w* = 12, *α*_1_ = *α*_2_ = 1, and *β*_1_ = *β*_2_ = 2; in (h), *w* = 13, *α*_1_ = *α*_2_ = 3, and *β*_1_ = *β*_2_ = 0; and in (i), *w* = 13, *α*_1_ = *α*_2_ = 3, and *β*_1_ = *β*_2_ = 2. In these simulations, (2) were numerically solved by employing the fourth-order Runge-Kutta integration method with integration step of 0.01. In addition, in all simulations, the initial condition is (*x*_1_(0), *y*_1_(0), *x*_2_(0), *y*_2_(0)) = (0,0, 0,0).

In (a) and (b), the populations are isolated. Therefore, the attractor can be either an equilibrium point or a limit cycle, because (2) split into two decoupled second-order systems. In fact, these are the attractors that can exist in the state space of second-order autonomous nonlinear systems [22–24].

In (a), the conditions for the asymptotical stability of the steady-state solution are satisfied (because *E* = 10.01 > *W* = 7.99 and *bc* = 200 > *EW*≃80.0). In this case, *x*_1_(*t*) converges to 0.167, which is the same value obtained from the analytical expression *x*_1_^*∗*^ = (*EI*_1_ − *bJ*_1_)/(*bc* − *EW*). Thus, the linear approximation of the sigmoidal function *S*(*z*) can be considered as valid to determine this equilibrium point and its stability. For *w* = *w*_*c*_ = 10.02, the system experiences a Hopf bifurcation. Hence, in (b), as *w* = 12 > *w*_*c*_, *x*_1_(*t*) tends to a periodic solution.

In cases (c)–(i), the populations are coupled. Now, the nature of the attractor is determined from its Lyapunov exponents *L*_1_, *L*_2_, *L*_3_, and *L*_4_, which were numerically computed by using the algorithm proposed by Wolf et al. [26]. When *L*_1,2,3,4_ < 0, the attractor is an equilibrium point; when *L*_1_ = 0 and *L*_2,3,4_ < 0, the attractor is a limit cycle; when *L*_1_ = *L*_2_ = 0 and *L*_3,4_ < 0, the attractor is a two-dimensional torus; when *L*_1_ > 0, *L*_2_ = 0, and *L*_3,4_ < 0, the attractor is chaotic [22–24].

In (c), the Routh-Hurwitz criterion is satisfied (because *γ*_1_ = 4.04 > 0, *γ*_2_≃243 > 0, *γ*_3_≃465, *γ*_4_≃14300 > 0, *δ*_1_≃128 > 0, and *δ*_2_≃13.6 > 0) and *x*_1_ converges to 0.175, which matches the number calculated from (3). In this case, *L*_1,2_≃−0.52 and *L*_3_≃*L*_4_ = −1.51.

As stated in Section 2, by varying the parameter values, if *δ*_2_ becomes equal to zero, then a Hopf bifurcation can take place. For instance, for *w* = 8 < *w*_*c*_ and *β*_1_ = *β*_2_ = 0, then *δ*_2_ = 0 if *α*_1_ = *α*_2_ = *α*_*c*_ = 2.02; thus, for *α*_1_ = *α*_2_ = 3 > *α*_*c*_, the attractor is a limit cycle, as shown in (d). In this case, *L*_1_≃0.00, *L*_2_≃−0.67, *L*_3_≃−1.48, and *L*_4_≃−3.32. Recall that *δ*_2_ = 0 is equivalent to *ϵ*_1_ ≡ (*w* + *α*)−(*a* + *d* + *e*) = 0 when *α*_1_ = *α*_2_ = *α* and *β*_1_ = *β*_2_ = *β*; thus, *ϵ*_1_ = 0 corresponds to *α*_*c*_ = *a* + *d* + *e* − *w* = 2.02, which is equal to the value numerically found. For *α* = *α*_*c*_, the oscillation period is about $2\pi /\sqrt{\epsilon_{2}}≃0.63$. In (d), for *α* = 3, the period is still 0.63.

Even when *w* = 8 < *w*_*c*_ and *α*_1_ = *α*_2_ = 1 < *α*_*c*_, a limit cycle arises if *β*_2_ > *β*_*c*_ = 2.25. Hence, in (e), with *β*_2_ = 3 > *β*_*c*_, the asymptotical behavior is a regular oscillation. It corresponds to a limit cycle, because *L*_1_≃0.00, *L*_2_≃−0.31, and *L*_3,4_≃−2.16.

It is easy to check that the steady state loses its stability when *W* > *E*, that is, when *w* > *w*_*c*_. In (f), with *w* = 12 > *w*_*c*_, *α*_1_ = *α*_2_ = 1, and *β*_1_ = *β*_2_ = 0, the trajectories in the state space converge to a two-dimensional torus. In fact, the Lyapunov exponents are *L*_1_≃*L*_2_≃0.00 and *L*_3_≃*L*_4_≃−0.55. Therefore, by increasing *w*, the system experiences a bifurcation concerning the birth of a toroidal attractor. In (g), by taking *β*_1_ = *β*_2_ = 2, the trajectories tend to a limit cycle, with *L*_1_≃0.00, *L*_2,3_≃−0.54, and *L*_4_≃−0.58. Thus, by increasing *β*_1_ and *β*_2_, the transition from torus towards cycle occurs.

In (h), for *w* = 13 > *w*_*c*_, *α*_1_ = *α*_2_ = 3 > *α*_*c*_, and *β*_1_ = *β*_2_ = 0, the attractor is chaotic, because *L*_1_≃0.28, *L*_2_≃0.00, *L*_3_≃−0.62, and *L*_4_≃−1.40. Observe that, when compared to (d), by increasing *w*, a transition from regular oscillation towards chaotic solution can occur. For these values of *α*_*i*_ and *β*_*i*_, there is chaos if 12.5≲*w*≲16.5. In (i), by taking *β*_1_ = *β*_2_ = 2, the attractor comes back to be a limit cycle, because *L*_1_≃0.00, *L*_2_≃−0.07, and *L*_3,4_≃−0.15. Thus, by increasing the values of *β*_1_ and *β*_2_, a bifurcation related to the transition from chaos to periodic behavior takes place.

Starting from a stationary solution, regular oscillations can be created by increasing *w* (please see (a) → (b) and (c) → (f)), or *α*_*i*_ (see (c) → (d)), or *β*_*i*_ (see (c) → (e)). From a periodic behavior, chaos can appear by a similar way (see (d) → (h)). Thus, by enhancing the excitatory connections, the activities of both populations can regularly or irregularly oscillate. However, when the strength of any excitatory connection is “too high,” the linear approximation of *S*(*z*) can no longer be valid. In this scenario, a suitable approximation is |*S*(*z*)|≃1 (because |*S*(*z*)| → 1 when |*z* | → *∞*). Therefore, from (2), |*x*_*i*_ | → 1/*a* and |*y*_*i*_ | → 1/*d* as the time passes. For instance, by simulating the system with *w* = 20, *α*_1_ = *α*_2_ = 3, and *β*_1_ = *β*_2_ = 0, the variables asymptotically converge to *x*_1_^*∗*^≃*x*_2_^*∗*^≃99.3 and *y*_1_^*∗*^≃*y*_2_^*∗*^≃98.5.

It is important to stress that the attractors and bifurcations reported in this section were already found in earlier works based on computer simulations on two-population models [10, 13–15]. We hope that the analytical and numerical results presented here can guide experimental researches on the detection of such attractors and bifurcations in actual neural networks, in particular the ones underlying working memory, as discussed in the next section.

## 4. Discussion

In this work, we explicitly derived analytical conditions concerning the stability of the steady state derived from the approximation *S*(*z*)≃*z*. Now, we discuss the possible relevance of this result.

Neural codes based on chaotic activity have been proposed [27–29]. We observed here that chaos can be found in a simple neuronal assembly composed of two coupled Wilson-Cowan populations subject to constant stimuli, when the strengths of the excitatory connections are above critical numbers. Since excitatory synapses are more often encountered than inhibitory ones [17], then these strengths can naturally be above such critical numbers. Thus, the irregular activity commonly found in EEG would be, at least partially, a simple consequence of the neuronal connectivity, in particular, the predominance of excitatory synapses.

The strengths of excitatory connections, however, cannot be “too high,” because in this case *S*(*z*)≃1 and the activity would tend to be stationary. Thus, the balance between excitation and inhibition must satisfy constraints assuring normal oscillatory behavior. Abnormalities in this balance in the cortical circuitry have been associated with neurological disorders, such as autism and epilepsy [30, 31].

The conditions presented in Section 2 can guide laboratory experiments, in order to verify their validity. Observe that these analytical expressions were derived by supposing that *S*(*z*)≃*z*. It is far from being trivial to find the regions in the 14-dimensional parameter space where this approximation holds. However, from (1) with *r* = 0, note that *dg*/*dt* = 0 implies *kg* ^*∗*^ = *S*(*z* ^*∗*^). Since $S(z)=z/\sqrt{1+z^{2}}≃z$ only if *z* ≪ 1, then *kg* ^*∗*^≃*z* ^*∗*^ ≪ 1 in the steady-state condition. Hence, in the numerical simulations, we took *a* = *d* = 0.01 ≪ 1 (obviously, *k* in (1) is equivalent to *a* and *d* in (2)). Neuronal populations with “slow” exponential decay (i.e., *a*, *d* ≪ 1) can be responsible for supporting working memory [32]. Hence, the validity of our conclusions could be first tested in such populations. Perhaps, these tests can reveal the true nature of the attractors and bifurcations involved in maintaining and manipulating new information.
